# Apparent Defective Abduction Without Diplopia

**DOI:** 10.7759/cureus.29155

**Published:** 2022-09-14

**Authors:** Danny Lam, Tyler R Blah, Fiona S Lau, Ashish Agar, Ian C Francis

**Affiliations:** 1 Ophthalmology, Royal Darwin Hospital, Darwin, AUS; 2 Ophthalmology, Royal North Shore Hospital, Sydney, AUS; 3 Ophthalmology, The Prince of Wales Hospital, Sydney, AUS

**Keywords:** saccades, diplopia, sixth cranial nerve palsy, neuro-ophthalmology, apparent defective abduction without diplopia

## Abstract

Sixth nerve palsies present with horizontal diplopia and typically have a neurological or neurovascular aetiology. They can be confirmed by clinically evaluating the velocity of the abducting saccade, which is slowed. Three cases are presented in which the patients had apparent defective abduction of one eye, resulting from not only neurological causes but also orbital causes. Clinicians should have a high index of suspicion in patients with defective abduction without diplopia and should include apparent defective abduction without diplopia (ADAD) in the list of potential differential diagnoses, considering not only neurological involvement but also orbital involvement.

## Introduction

The sixth cranial nerve is responsible for the innervation of the lateral rectus muscle, which abducts the eye. Regardless of the mechanism, incomplete or complete sixth nerve deficits result in ipsilateral abduction deficiency and are present with horizontal diplopia [1]. Horizontal diplopia for distance, with the patient being esophoric or esotropic for distance and orthophoric for near, suggests an ipsilateral sixth nerve palsy.

Traditionally, neurological diagnosis has suggested that if more conjunctiva/sclera is seen when a patient attempts to abduct an eye, an ipsilateral sixth nerve palsy may be present. However, there are other well-recognised causes of defective abduction of an eye, including horizontal gaze palsy, medial blowout fracture, chronic progressive external ophthalmoplegia, orbital myositis, lymphoma in an extraocular muscle, orbital involvement by benign and malignant tumours, orbital fibrosis syndrome, Duane's syndrome, myasthenia gravis, thyroid orbitopathy, and orbital inflammatory syndrome.

Sixth nerve palsy may be classified as either isolated or non-isolated, whereby the cause of an isolated sixth nerve palsy is frequently associated with congenital, traumatic, post-viral, microvascular, or idiopathic aetiologies [2-4]. Clinical presentation typically includes horizontal diplopia which worsens with a horizontal gaze toward the side of the paretic lateral rectus muscle [1,5]. In the correct context, a sixth nerve palsy can be confirmed by clinically evaluating the velocity of the abducting saccade, which is slowed [6]. In almost all cases of sixth nerve palsy, a neurological or neurovascular aetiology can be identified, with the most common cause being presumed microvascular ischemia. Also, the sixth nerve palsy's neuroanatomical and neurological characteristics usually make it possible to make a convincing and localising diagnosis.

However, neurological diagnosis of defective eye movements should not rely on the amount of sclera visible during an attempted abduction of the eye. Furthermore, there is no study on scleral visibility in patients with apparent defective abduction, as a small amount of scleral visibility on abduction can be normal.

In this report, the authors document two patients who had apparently defective abduction of one eye on attempted abduction. However, these patients demonstrated that the suspected sixth nerve palsy was in fact apparent defective abduction without diplopia (ADAD). In both of these patients, the recognition of ADAD was helpful diagnostically and therapeutically.

## Case presentation

Case 1

A 62-year-old woman was diagnosed with meningiomas involving both the left greater wing of the sphenoid ridge and the right anterior cranial fossa. Three years later, she presented for ophthalmological review as part of ongoing monitoring and multidisciplinary care. During this time, she remained stable during the management of her tumours.

Following de-bulking radiation therapy one year after diagnosis, examination revealed 4 mm of left proptosis and mild optic nerve pallor. Fortunately, Humphrey Visual Field (HVF) testing showed no abnormalities.

Two years after diagnosis, in a combined neurosurgical and orbital surgical procedure, she underwent successful de-bulking of the left sphenoid wing lesion. One year later, magnetic resonance imaging (MRI) performed showed no enlargement of her sphenoid wing meningioma. She had experienced mild left facial (V2 distribution) pain, unchanging over 6-12 months, managed with paracetamol. On examination, her facial sensation and ocular rotations (OR) were normal.

**Figure 1:**
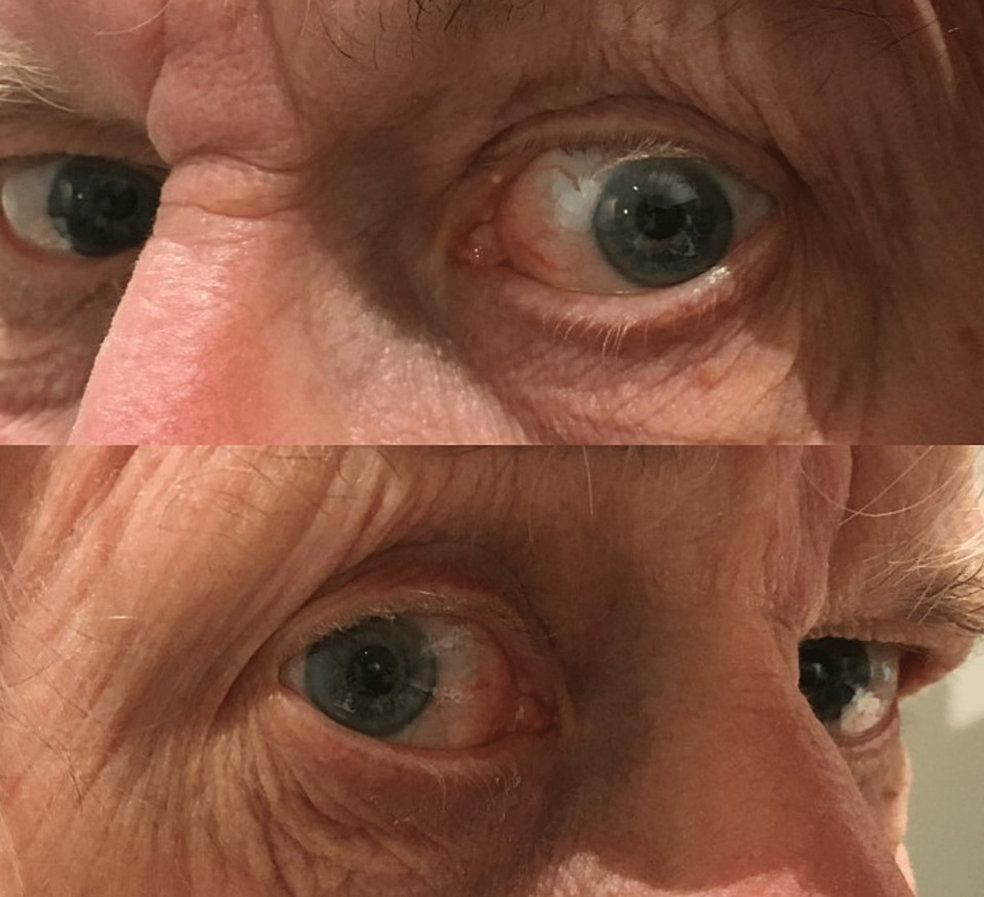
Left eye apparent defective abduction (top) compared with right eye abduction (bottom)

Three years after the presentation she demonstrated visual acuity of 6/9 in the right eye and 6/12 in the left, explained by bilateral cataracts. She had a grade 2/4 left relative afferent pupillary defect (0.9 log units using the Neutral Density Filter), with mild left optic atrophy. Cranial nerve examination was otherwise normal, with the exception of apparent mild defective abduction of the left eye. There was more sclera visible in the abduction of the left eye compared with the abduction of the right eye (Figure 1). Despite this, she had no diplopia in horizontal gaze, and cover testing for distance (CTD) and near (CTN) was normal. Further, the velocity of her left eye abducting saccade was normal [7]. Based on the history of left orbital surgery and no other confirmatory features of sixth nerve palsy, it was therefore likely that the patient’s apparent defective abduction was related to the orbital surgery. The authors have labelled this ADAD.

The patient’s orbital and meningioma status were kept under routine neurosurgical, ophthalmological, and radiation oncology review.

Case 2

A 53-year-old woman was referred by her primary care physician for the management of her thyroid orbitopathy. Despite her disorder, the patient continued to smoke 10 cigarettes per day.

On examination, her vision was 6/6 in both eyes, with normal colour vision on Farnsworth-Munsell testing. The HVF was normal in the right eye, with minimal changes in the left visual field. There was marked increased bilateral orbital resiliency, confirmed by reduced retropulsion [7]. Her cranial nerves were otherwise normal.

Her intraocular pressures in primary gaze were 22 mmHg bilaterally by Goldmann Applanation Tonometry, elevating on upgaze to 36 mmHg in the right eye and 35 mmHg in the left, a positive Braley’s sign [8]. Mild right Superior Limbic Keratoconjunctivitis of Theodore and a small amount of fluorescein staining on the left inferior cornea were noted. She demonstrated defective abduction of each eye but without diplopia, and the sclera and conjunctiva were visible in both eyes on attempted abduction. Her ocular rotations were normal, and the velocity of the abducting saccade in each eye was normal. This was considered a further case of ADAD.

Three months later, following the cessation of smoking and a medial and lateral wall orbital decompression, the patient had 6/4 corrected vision bilaterally, normal HVFs, colour vision, and pupils. Notably, her thyroid orbitopathy had stabilised. The review was planned for 12 months.

## Discussion

The authors have documented two patients who had apparent defective abduction without diplopia of one eye, as there was greater visibility of the temporal conjunctiva and sclera on attempted abduction. However, in each case, an orbital explanation was able to demonstrate that this represented ADAD. This was confirmed by the fact that CTN, CTD, OR, and abducting saccadic velocity were all normal. Therefore, ADAD appears to be a mechanism that appropriately explains these presentations.

However, defective abduction without diplopia is more commonly associated with cases of longstanding Duane’s syndrome, congenital strabismus, combined third and sixth palsies, Hoyt-Unsöld syndrome, in patients with cognitive dysfunction, and in those with a non-organic basis of their symptomatology. Identification of an aetiology is appropriately achieved by combining a definitive history with cover testing, testing of OR, and saccadic velocity testing.

ADAD occurs when there is obvious conjunctiva and sclera visible in the ‘affected’ eye on attempted abduction. Indeed, some clinicians consider that this represents defective abduction. The precise measurement of the horizontal distances between the lateral canthus, pupil midline, medial canthus, and facial midline can help to confirm the diagnosis of ADAD. The techniques mentioned above appear adequate for making the appropriate diagnosis.

Clinicians should be aware that observing an asymmetric degree of white in the eye when the eye is abducted does not mandate that there is defective abduction, let alone a sixth nerve palsy. While a patient who demonstrates defective abduction of one eye may indeed have a genuine sixth nerve palsy, the associated history and examination findings will confirm or deny this, and ADAD may readily explain this defective abduction.

Furthermore, it is imperative that the clinician bear in mind that neurological principles suggest that if there is defective abduction and diplopia, then there may well be a paretic muscle (the lateral rectus muscle in this case). This will be categorically confirmed by the associated slow abducting saccade. Moreover, orbital examination principles suggest that if there is an orbital mass with associated defective abduction and diplopia, any sixth nerve palsy will be confirmed by a slow velocity abducting saccade.

Therefore, the causes of defective abduction with no diplopia (excluding gaze palsies) should include ADAD, and this observation indeed may be common. However, ADAD remains underdiagnosed because patients with neurological dysfunction may have ADAD but not necessarily sixth nerve palsy.

## Conclusions

The authors have described the phenomenon of ADAD. Clinicians should have a high index of suspicion in patients with defective abduction without diplopia. They should rely on considering not only neurological aetiology but also the possibility that orbital or eyelid surgery may have produced ADAD, which improves the diagnostic status of these patients.

## References

[1] Elder C, Hainline C, Galetta SL, Balcer LJ, Rucker JC (2016). Isolated abducens nerve palsy: update on evaluation and diagnosis. Curr Neurol Neurosci Rep.

[2] Mutyala S, Holmes JM, Hodge DO, Younge BR (1996). Spontaneous recovery rate in traumatic sixth-nerve palsy. Am J Ophthalmol.

[3] Werner DB, Savino PJ, Schatz NJ (1983). Benign recurrent sixth nerve palsies in childhood. Secondary to immunization or viral illness. Arch Ophthalmol.

[4] Lee MS, Galetta SL, Volpe NJ, Liu GT (1999). Sixth nerve palsies in children. Pediatr Neurol.

[5] Wilker SC, Rucker JC, Newman NJ, Biousse V, Tomsak RL (2009). Pain in ischaemic ocular motor cranial nerve palsies. Br J Ophthalmol.

[6] Ling ML, Tynan D, Ruan CW, Lau FS, Spencer SK, Agar A, Francis IC (2020). Assessment of saccadic velocity at the bedside. Neuroophthalmology.

[7] Lee BW, Ling ML, Francis IC (2019). Re: “Accuracy of simple quantitative assessment of orbital resiliency”. Ophthalmic Plast Reconstr Surg.

[8] Braley AE (1953). Malignant exophthalmos. Am J Ophthalmol.

